# Cardiovascular toxicity profiles of immune checkpoint inhibitors with or without angiogenesis inhibitors: a real-world pharmacovigilance analysis based on the FAERS database from 2014 to 2022

**DOI:** 10.3389/fimmu.2023.1127128

**Published:** 2023-05-24

**Authors:** Yanfeng Wang, Chanjuan Cui, Lei Deng, Lin Wang, Xiayang Ren

**Affiliations:** ^1^ Department of Comprehensive Oncology, National Cancer Center/National Clinical Research Center for Cancer/Cancer Hospital, Chinese Academy of Medical Sciences and Peking Union Medical College, Beijing, China; ^2^ Department of Laboratory Medicine, National Cancer Center/National Clinical Research Center for Cancer/Cancer Hospital, Chinese Academy of Medical Sciences and Peking Union Medical College, Beijing, China; ^3^ Department of Radiation Oncology, National Cancer Center/National Clinical Research Center for Cancer/Cancer Hospital, Chinese Academy of Medical Sciences and Peking Union Medical College, Beijing, China; ^4^ Department of Medical Oncology, National Cancer Center/National Clinical Research Center for Cancer/Cancer Hospital, Chinese Academy of Medical Sciences and Peking Union Medical College, Beijing, China; ^5^ Department of Pharmacy, National Cancer Center/National Clinical Research Center for Cancer/Cancer Hospital, Chinese Academy of Medical Sciences and Peking Union Medical College, Beijing, China

**Keywords:** cardiovascular toxicity, immune checkpoint inhibitor, angiogenesis inhibitor, combination therapy, FAERS database, disproportionality analysis

## Abstract

**Background:**

Immune checkpoint inhibitors (ICIs) combined with angiogenesis inhibitors (AGIs) have become increasingly available for multiple types of cancers, although the cardiovascular safety profiles of this combination therapy in real-world settings have not been elucidated to date. Therefore, we aimed to comprehensively investigate the cardiovascular toxicity profiles of ICIs combined with AGIs in comparison with ICIs alone.

**Methods:**

The Food and Drug Administration Adverse Event Reporting System (FAERS) database from the 1^st^ quarter of 2014 to the 1^st^ quarter of 2022 was retrospectively queried to extract reports of cardiovascular adverse events (AEs) associated with ICIs alone, AGIs alone and combination therapy. To perform disproportionality analysis, the reporting odds ratios (RORs) and information components (ICs) were calculated with statistical shrinkage transformation formulas and a lower limit of the 95% confidence interval (CI) for ROR (ROR_025_) > 1 or IC (IC_025_) > 0 with at least 3 reports was considered statistically significant.

**Results:**

A total of 18 854 cardiovascular AE cases/26 059 reports for ICIs alone, 47 168 cases/67 595 reports for AGIs alone, and 3 978 cases/5 263 reports for combination therapy were extracted. Compared to the entire database of patients without AGIs or ICIs, cardiovascular AEs were overreported in patients with combination therapy (IC_025_/ROR_025_ = 0.559/1.478), showing stronger signal strength than those taking ICIs alone (IC_025_/ROR_025_ = 0.118/1.086) or AGIs alone (IC_025_/ROR_025_ = 0.323/1.252). Importantly, compared with ICIs alone, combination therapy showed a decrease in signal strength for noninfectious myocarditis/pericarditis (IC_025_/ROR_025_ = 1.142/2.216 *vs*. IC_025_/ROR_025_ = 0.673/1.614), while an increase in signal value for embolic and thrombotic events (IC_025_/ROR_025_ = 0.147/1.111 *vs*. IC_025_/ROR_025_ = 0.591/1.519). For outcomes of cardiovascular AEs, the frequency of death and life-threatening AEs was lower for combination therapy than ICIs alone in noninfectious myocarditis/pericarditis (37.7% *vs*. 49.2%) as well as in embolic and thrombotic events (29.9% *vs*. 39.6%). Analysis among indications of cancer showed similar findings.

**Conclusion:**

Overall, ICIs combined with AGIs showed a greater risk of cardiovascular AEs than ICIs alone, mainly due to an increase in embolic and thrombotic events while a decrease in noninfectious myocarditis/pericarditis. In addition, compared with ICIs alone, combination therapy presented a lower frequency of death and life-threatening in noninfectious myocarditis/pericarditis and embolic and thrombotic events.

## Introduction

Over the past decade, immune checkpoint inhibitors (ICIs), including programmed death-1 (PD-1) inhibitors, programmed death ligand-1 (PD-L1) inhibitors and cytotoxic T-lymphocyte antigen-4 (CTLA-4) inhibitors, have revolutionized the therapeutic strategy in a wide variety of tumours (1). Regrettably, the clinical benefit for ICIs alone was limited, and effectiveness was not observed in some patients due to resistance. To improve the treatment efficacy, several novel combination approaches have been developed. Recently, a growing body of evidence has revealed that angiogenesis inhibitors (AGIs) targeting the vascular endothelial growth factor (VEGF) signalling pathway, mainly including anti-VEGF monoclonal antibodies (mAbs), anti-VEGF receptor (VEGFR) mAbs, VEGF soluble decoy receptor capturing free available VEGF (VEGF-trap), and tyrosine kinase inhibitors (TKIs) with anti-VEFGR activity, combined with ICIs can exert synergistic anti-tumour effects against some solid tumours, such as renal cell carcinoma (RCC), non-small cell lung cancer (NSCLC), hepatocellular carcinoma (HCC), endometrial cancer and melanoma. (2–9). However, the combination approach is expected to be accompanied by an increase in cardiovascular adverse events (AEs), as both agents have been established to be associated with specific cardiovascular AEs, such as myocarditis for ICIs (10, 11), hypertension, embolic and thrombotic events for AGIs (12). However, the cardiovascular safety profiles of this combination therapy in real-world settings have not been well elucidated to date (13, 14).

Therefore, we aimed to comprehensively investigate the cardiovascular toxicity profiles for ICIs in combination with AGIs in terms of frequency, spectrum and outcomes to provide new insights into the cardiovascular safety profiles of combination therapy.

## Materials and methods

### Study design and data sources

This retrospective pharmacovigilance analysis is based on the United States (US) Food and Drug Administration (FDA) Adverse Event Reporting System (FAERS) database, which is a publicly available postmarketing safety surveillance database containing millions of real-world spontaneous AE cases/reports submitted by healthcare professionals, individual patients and drug manufacturers. The large quantity of the data collected at a national level from a large population around the world makes FAERS robust for conducting pharmacovigilance studies in the real-world settings.

In the FAERS, AEs are coded using the preferred terms (PTs) according to the Medical Dictionary for Regulatory Activities (MedDRA) (Version 24.0), and all PTs representing symptoms, signs, and investigations likely to be relevant can be grouped into narrative categories using the Standardized MedDRA Queries (SMQs) to define a medical condition of interest. In this study, cardiovascular AEs were grouped into 9 narrow categories of SMQs (cardiac arrhythmia, cardiac failure, cardiomyopathy, embolic and thrombotic events, hypertension, ischaemic heart disease, noninfectious myocarditis/pericarditis, pulmonary hypertension, and torsade de pointes/QT prolongation) (see **Table 1**; **Tables S1–S9** in **Supplementary Materials**).

**Table 1:** Cardiovascular adverse events grouped into 9 narrow categories of Standardized MedDRA Queries (SMQs) according to MedDRA 24.0.

SMQ name	SMQ code	Algorithm
Cardiac arrhythmias	20000049	Narrow
Cardiac failure	20000004	Narrow
Cardiomyopathy	20000150	Narrow
Embolic and thrombotic events	20000081	Narrow
Hypertension	20000147	Narrow
Ischaemic heart disease	20000043	Narrow
Noninfectious myocarditis/pericarditis	20000239	Narrow
Pulmonary hypertension	20000130	Narrow
Torsade de pointes/QT prolongation	20000001	Narrow

### Data extraction

**Figure 1:**
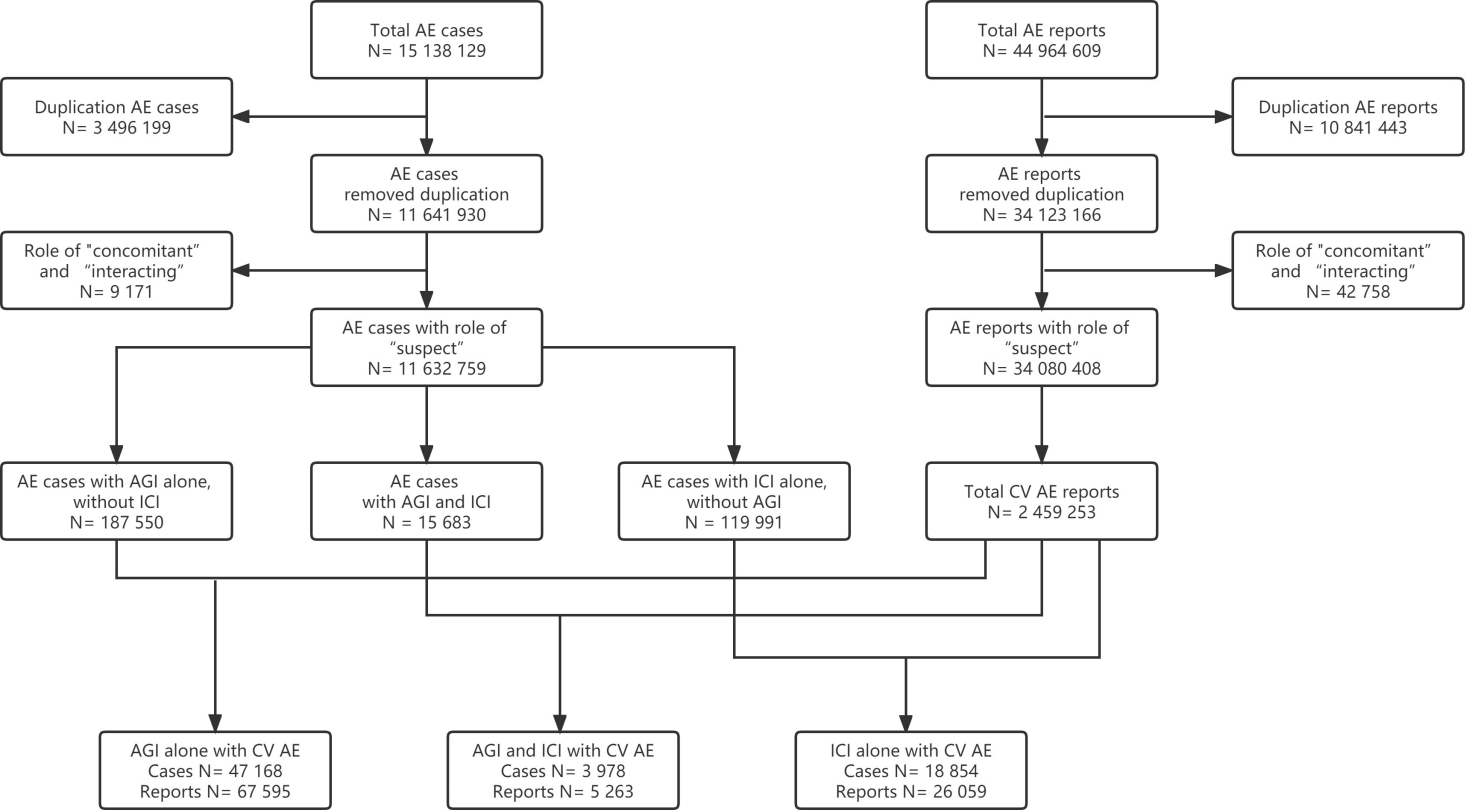
Flow chart of cardiovascular adverse event (AE) cases and reports selection for AGI alone, ICI combined with AGI, and ICI alone (AE, adverse event; AGI, angiogenesis inhibitor; CV, cardiovascular; ICI, immune checkpoint inhibitor).

This study collected data from the FAERS database covering the period from the 1^st^ quarter of 2014 to the 1^st^ quarter of 2022. In the FAERS database, a reported AE case (patient) may have more than one AE report. Thus, three-step data cleaning was conducted before analysis (**Figure 1**). Firstly, duplicates (the same case/report submitted by different sources) and multiple cases/reports (follow-ups of the same case/report with additional and updated information) were removed, and only the most recent version of each case/report without duplication was extracted. Secondly, only AE cases/reports with reported roles of drugs as “suspect” were included, while those with roles of “concomitant” or “interacting” were removed. Finally, a drug event combination (DEC) was established by combining cardiovascular AE cases/reports based on three subgroups, that was, ICIs alone without AGIs, AGIs alone without ICIs, and ICIs combined with AGIs.

Notably, the drugs are reported as free text in FAERS, either generic names or brand names, even research codes can be reported, and misspelling can also be present. Thus, a thorough drug name archive including all generic names, brand names and research codes of ICIs and AGIs approved by the US FDA or the National Medical Products Administration (NMPA) in China (formerly known as China Food and Drug Administration, CFDA) as of December 31, 2021, was applied (see **Table S10** in **Supplementary Materials**).

### Statistical analysis

Currently, disproportionality analysis (also known as case-noncase analysis) is a widely used signal detection method in pharmacovigilance studies based on a two-by-two contingency table (**Table 2**) (15, 16). The reporting odds ratio (ROR) and information component (IC) are two specific indices calculated to detect potential associations between drugs and AEs. Notably, statistical shrinkage transformation was applied to obtain robust results and the corresponding calculation formulas for ROR and IC are as follows (17):

**Table 2:** Disproportionality analysis based on two-by-two contingency table.

	Target adverse events	Other adverse events	Total
Target drug	a (N_observed_)	b	N_drug_=a+b
Other drugs	c	d	c+d
Total	N_event_=a+c	b+d	N_total_=a+b+c+d


$$\text{ROR}=\left({\text{N}}_{\text{observed}}+0.5\right)/\left({\text{N}}_{\text{expected}}+0.5\right)$$

$$\text{IC}=\log_{2}\left[\left({\text{N}}_{\text{observed}}+0.5\right)/\left({\text{N}}_{\text{expected}}+0.5\right)\right]$$

$${\text{N}}_{\text{expected}}={\text{N}}_{\text{drug}}*{\text{N}}_{\text{event}}/{\text{N}}_{\text{total}}$$

where N_observed_ (a) is the observed number of reports of target drug AEs, N_expected_ is the expected number of reports of target drug AEs, N_drug_ (a+b) is the total number of reports of a target drug, N_event_ (a+c) is the total number of reports of target AEs, and N_total_ (a+b+c+d) is the total number of reports in the entire database.

Moreover, the calculation formulas for the 95% confidence intervals (CIs) of ROR and IC are as follows:


$$\text{ROR }95\text{\%CI}=e^{ln\left(ROR\right)\pm 1.96\sqrt{\frac{1}{a}+\frac{1}{b}+\frac{1}{c}+\frac{1}{d}}}$$

$${\text{IC}}_{025}=\text{IC}-3.3*{\left({\text{N}}_{\text{observed}}+0.5\right)}^{-0.5}-2*{\left({\text{N}}_{\text{observed}}+0.5\right)}^{-1.5}$$

$${\text{IC}}_{075}=\text{IC}+2.4*{\left({\text{N}}_{\text{observed}}+0.5\right)}^{-0.5}-0.5*{\left({\text{N}}_{\text{observed}}+0.5\right)}^{-1.5}$$

Generally, a lower limit of the 95%CI for ROR (ROR_025_) > 1 or a lower limit of the 95%CI for IC (IC_025_) > 0 with at least 3 reports was considered statistically significant and deemed a potential signal.

All analyses were performed using SAS version 9.4 (SAS Institute Inc., Cary, NC, United States).

## Results

### Descriptive analysis

From the 1^st^ quarter of 2014 to the 1^st^ quarter of 2022, a total of 15 138 129 AE cases and a total of 44 964 609 AE reports were extracted from the database. After the data cleaning, 18 854 cardiovascular AE cases and 26 059 reports for ICIs alone without AGIs, 47 168 AE cases and 67 595 reports for AGIs alone without ICIs, and 3 978 cases and 5 263 reports for ICIs combined with AGIs were included for the final analysis (**Figure 1**).

Baseline characteristics of patients (cases) with cardiovascular AEs reported for AGIs alone, ICIs alone and combination therapy are presented and compared in **Table 3**. The patient ages were comparable among these three groups, while a higher percentage of females was reported in the combination therapy group, which may be due to more reports associated with endometrial cancer.

**Table 3:** Baseline characteristics of cardiovascular reports associated with angiogenesis inhibitor, immune checkpoint inhibitor and combination therapy from 2014 Q1 to 2022 Q1.

Characteristics	Angiogenesis inhibitor (n = 47 168)	Immune checkpoint inhibitor (n = 18 854)	Combination therapy (n = 3 978)
Patient's age			
Data available [n (%) ]	34 423 (73.0)	15 019 (79.6)	3 150 (79.2)
Age, years, median (Q1-Q3)	66 (57, 73)	67 (58, 73)	66 (58, 73)
Age group [n (%) ]			
< 18 years	660 (1.9)	334 (2.2)	118 (3.7)
18-65 years	15 260 (44.3)	6 141 (40.9)	1 250 (39.7)
≥ 65 years	18 503 (53.8)	8 544 (56.9)	1 782 (56.6)
Unknown	12 745 (27.0)	3 835 (20.4)	828 (20.8)
Patient's gender [n (%) ]			
Male	23 986 (50.9)	11 014 (58.4)	1 930 (48.5)
Female	18 539 (39.3)	6 451 (34.2)	1 793 (45.1)
Unknown	4 643 (9.8)	1 389 (7.4)	255 (6.4)
Type of reporter			
Health professional	29 886 (63.4)	14 170 (75.1)	2 965 (74.5)
Non-health professional	16 496 (35.0)	4 466 (23.7)	1 002 (25.2)
Unknown	786 (1.6)	218 (1.2)	11 (0.3)
Outcome of adverse events			
Death	8 131 (17.2)	5 118 (27.1)	666 (16.7)
Life-threatening	1 761 (3.7)	1 302 (6.9)	112 (2.8)
Caused/prolonged hospitalization	14 797 (31.4)	6 753 (35.8)	1313 (33.0)
Disabling/incapacitating	238 (0.5)	94 (0.5)	11 (0.3)
Congenital anomaly	1 (0.0)	3 (0.0)	0 (0.0)
Other serious events	12 901 (27.4)	3 443 (18.3)	631 (15.9)
Unknown	9 339 (19.8)	2 141 (11.4)	1 245 (31.3)
Reported indication			
Non-small cell lung cancer	1 475 (3.1)	4 806 (25.5)	453 (11.4)
Renal cell carcinoma	9 727 (20.6)	1 429 (7.6)	1 117 (28.1)
Hepatocellular carcinoma	3 121 (6.6)	150 (0.8)	478 (12.0)
Colorectal cancer	6 439 (13.6)	265 (1.4)	91 (2.3)
Endometrial cancer	309 (0.6)	66 (0.4)	665 (16.7)
Breast cancer	1 443 (3.0)	414 (2.2)	22 (0.6)
Melanoma	88 (0.2)	3 105 (16.5)	87 (2.2)
Reported countries			
United States	23 126 (49.0)	6 368 (33.8)	1 795 (45.1)
Canada	1 980 (4.2)	896 (4.8)	133 (3.3)
Great Britain	1 316 (2.8)	570 (3.0)	156 (3.9)
Germany	2 072 (4.4)	1 298 (6.9)	205 (5.2)
France	2 287 (4.8)	1 859 (9.8)	260 (6.5)
Italy	1 571 (3.3)	608 (3.2)	53 (1.3)
Japan	5 254 (11.1)	3 015 (16.0)	610 (15.4)
China	1 167 (2.5)	472 (2.5)	111 (2.8)
Other countries	8 307 (17.5)	3 740 (19.9)	650 (16.4)
Unknown	88 (0.2)	28 (0.1)	5 (0.1)
Reported year			
2014	5 203 (11.0)	242 (1.3)	1 (0.0)
2015	6 200 (13.1)	588 (3.1)	17 (0.4)
2016	4 712 (10.0)	1 311 (7.0)	53 (1.3)
2017	5 029 (10.7)	2 177 (11.5)	111 (2.8)
2018	5 861 (12.4)	2 696 (14.3)	197 (5.0)
2019	5 635 (11.9)	3 294 (17.5)	354 (8.9)
2020	5 851 (12.4)	3 535 (18.7)	871 (21.9)
2021	5 576 (11.8)	3 115 (16.5)	1 304 (32.8)
2022 **Q1**	3 101 (6.7)	1 896 (10.0)	1 070 (26.9)

Regarding the reported indications, the most frequently reported types of cancers were RCC (20.6%), colorectal cancer (CRC) (13.6%) and HCC (6.6%) for AGIs alone; NSCLC (25.5%), melanoma (16.5%) and RCC (7.6%) for ICIs alone; and RCC (28.1%), endometrial cancer (16.7%), HCC (12.0%) and NSCLC (11.4%) for combination therapy.

Notably, with respect to chronological trends, the proportion of reported cardiovascular AEs for AGIs alone did not change significantly from 2014 to 2021, ranging from 10.0% to 12.4%; the reported proportion for ICIs alone gradually increased from 2014 to 2019 (from 1.3% to 17.5%) and then remained relatively stable from 2019 to 2021 (from 16.5% to 18.7%); and the reported proportion for combination therapy increased year by year from 2014 to 2019 (from 0.0% to 8.9%), with a rapid increase from 2019 to 2021 (from 8.9% to 32.8%).

### Disproportionality analysis for AGIs alone, ICIs alone and combination therapy

Overall, compared to the entire database without AGIs or ICIs, cardiovascular AEs were overreported in patients using AGIs alone (IC_025_/ROR_025_ = 0.323/1.252), ICIs alone (IC_025_/ROR_025_ = 0.118/1.086), and combination therapy (IC_025_/ROR_025_ = 0.559/1.478), showing stronger signal strength for combination therapy (**Table 4**).

**Table 4:** Overall disproportionality analysis for angiogenesis inhibitor, immune checkpoint inhibitor, and combination therapy.

Class of agent	N	IC	IC_025_	IC_975_	ROR	ROR_025_	ROR_975_
AGI alone without ICI	67 595	0.336	0.323	0.345	1.262	1.252	1.272
ICI alone without AGI	26 059	0.138	0.118	0.153	1.100	1.086	1.114
Combination therapy	5 263	0.605	0.559	0.638	1.521	1.478	1.565

AGI, angiogenesis inhibitor; IC, information component; ICI, immune checkpoint inhibitor; ROR, reporting odds ratio.

Of note, 109, 102 and 59 PTs were detected as signals for AGIs alone, ICIs alone and combination therapy, respectively, based on the IC_025_>0 criterion, while 120, 109 and 70 PTs were detected as signals based on the ROR_025_>1 criterion, respectively (see **Table S11** in **Supplementary Materials**). Interestingly, compared with ICIs alone, combination therapy depicted a decrease in both IC_025_ and ROR_025_ for some specific PTs, including immune-mediated myocarditis (IC_025_ from 6.103 to 4.107, ROR_025_ from 35.596 to 12.092), autoimmune myocarditis (IC_025_ from 5.429 to 2.945, ROR_025_ from 36.160 to 8.052) and myocarditis (IC_025_ from 4.387 to 3.955, ROR_025_ from 20.961 to 15.920). In contrast, in comparison to AGIs alone, combination therapy demonstrated an increase for some PTs such as embolism (IC_025_ from 2.061 to 3.120, ROR_025_ from 4.217 to 9.028) and portal vein thrombosis (IC_025_ from 2.136 to 2.907, ROR_025_ from 4.463 to 7.958).

**Figure 2:**
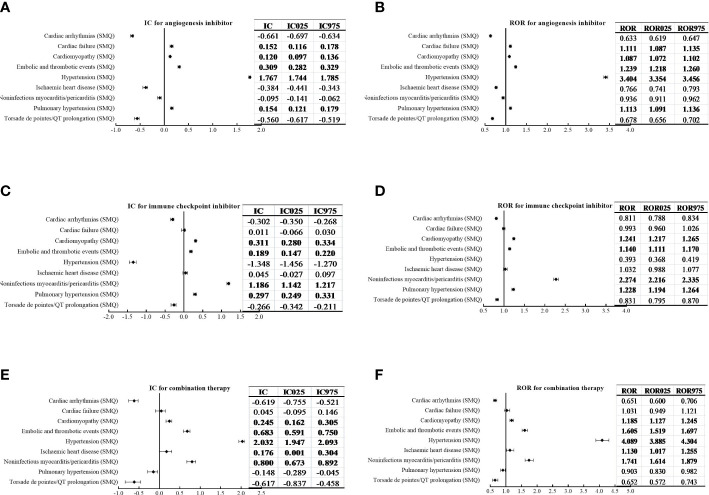
Comparison of the cardiovascular adverse events based on the Standardized Medical Dictionary for Regulatory Activities (MedDRA) Queries (SMQs) for angiogenesis inhibitors **(A, B)**, immune checkpoint inhibitors **(C, D)**, and combination therapy **(E, F)** with the information component (IC) and reporting odds ratio (ROR) (IC, information component; ROR, reporting odds ratio).

Furthermore, based on specific SMQs of cardiovascular AEs, using AGIs alone was significantly associated with cardiac failure (SMQ), cardiomyopathy (SMQ), embolic and thrombotic events (SMQ), hypertension (SMQ) and pulmonary hypertension (SMQ), with the strongest signal value for hypertension (SMQ) (IC_025_/ROR_025_ = 1.744/3.354); using ICIs alone was associated with cardiomyopathy (SMQ), embolic and thrombotic events (SMQ), noninfectious myocarditis/pericarditis (SMQ) and pulmonary hypertension (SMQ), with the strongest signal value for noninfectious myocarditis/pericarditis (SMQ) (IC_025_/ROR_025_ = 1.142/2.216); and combination therapy was associated with cardiomyopathy (SMQ), embolic and thrombotic events (SMQ), hypertension (SMQ), ischaemic heart disease (SMQ) and noninfectious myocarditis/pericarditis (SMQ), with the strongest signal value for hypertension (SMQ) (IC_025_/ROR_025_ = 1.947/3.885). Similarly, combination therapy showed a decrease in signal strength for noninfectious myocarditis/pericarditis (SMQ) compared with ICIs alone (IC_025_ = 0.673 *vs*. IC_025_ = 1.142; ROR_025_ = 1.614 *vs*. ROR_025_ = 2.216), whereas there was an increase in signal value for embolic and thrombotic events (SMQ) (IC_025_/ROR_025_ = 0.591/1.519) compared with AGIs alone (IC_025_/ROR_025_ = 0.282/1.218) or ICIs alone (IC_025_/ROR_025_ = 0.147/1.111) (**Figures 2A–F**).

### Outcomes of cardiovascular adverse events

As shown in **Table 3**, the most serious cardiovascular AEs, namely, death and life-threatening AEs, were reported with the highest frequency for ICIs alone (27.1% and 6.9%), followed by AGIs alone (17.2% and 3.7%) and combination therapy (16.7% and 2.8%).

**Figure 3:**
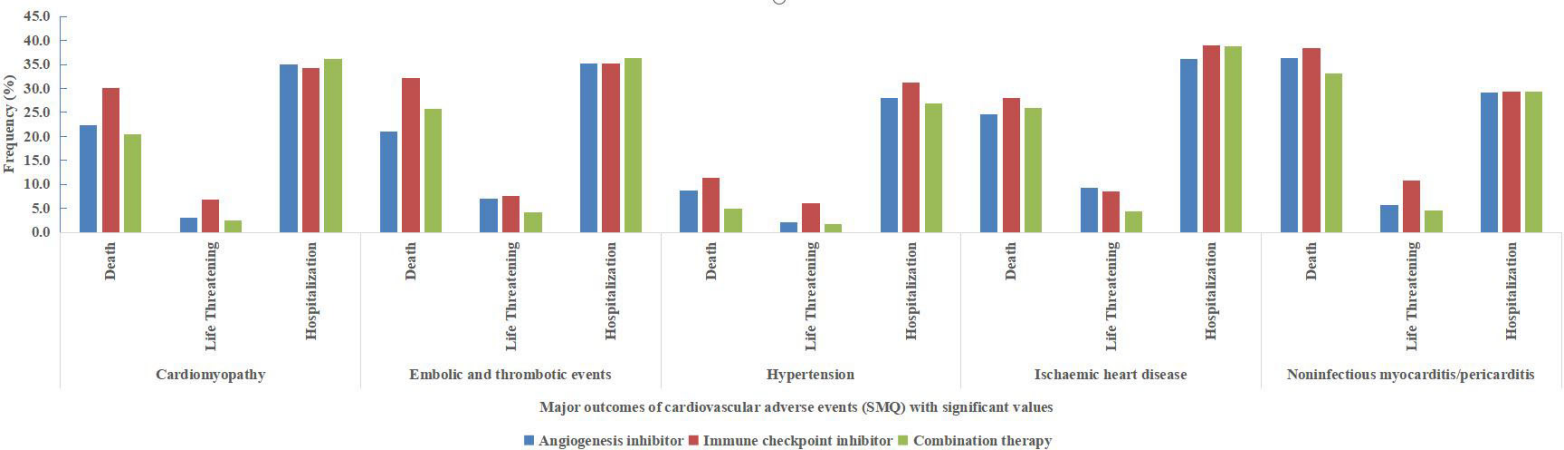
Major outcomes (death, life-threatening and hospitalization) of cardiovascular adverse events based on the Standardized Medical Dictionary for Regulatory Activities (MedDRA) Queries (SMQs) with significant values (cardiomyopathy, embolic and thrombotic events, hypertension, ischaemic heart disease and noninfectious myocarditis/pericarditis).

Furthermore, the outcomes of cardiovascular AEs based on specific SMQs including cardiomyopathy (SMQ), embolic and thrombotic events (SMQ), hypertension (SMQ), ischaemic heart disease (SMQ), and noninfectious myocarditis/pericarditis (SMQ), which were detected as significant signals are depicted in **Figure 3**.

Notably, compared with ICIs alone, combination therapy showed a lower frequency of death and life-threatening events in all 5 cardiovascular AEs based on SMQs. Furthermore, in noninfectious myocarditis/pericarditis (SMQ), the frequency of death and life-threatening events was 38.4% and 10.8% (accounting for 49.2%) for ICI alone, and 33.2% and 4.5% (accounting for 37.7%) for combination therapy, respectively. Moreover, in embolic and thrombotic events (SMQ), the frequency of death and life-threatening events was 39.6% for ICIs alone, and 29.9% for combination therapy.

### Further analysis among indications of malignant tumours

**Figure 4:**
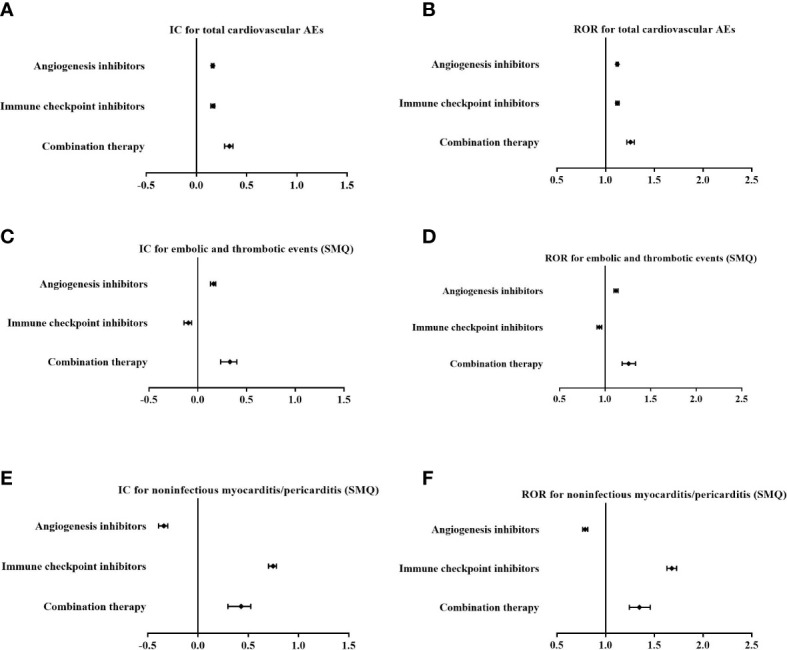
Comparison of overall cardiovascular adverse events (AEs) **(A, B)**, embolic and thrombotic events (SMQ) **(C, D)** and noninfectious myocarditis/pericarditis (SMQ) **(E, F)** for angiogenesis inhibitors alone, immune checkpoint inhibitors alone, and combination therapy among cases with indications of malignant tumours with the information component (IC) and reporting odds ratio (ROR) (IC, information component; ROR, reporting odds ratio).

Among AE cases with indications of malignant tumours, disproportionality analysis also showed that cardiovascular AEs were overreported in patients using combination therapy (IC_025_/ROR_025_ = 0.280/1.218) with a stronger signal strength than those using AGIs alone (IC_025_/ROR_025_ = 0.148/1.108) or ICIs alone (IC_025_/ROR_025_ = 0.144/1.106) (**Figures 4A, B**).

In addition, based on specific SMQs of cardiovascular AEs, there was an increase in the signal value for embolic and thrombotic events (SMQ) in combination therapy (IC_025_/ROR_025_ = 0.235/1.187) compared with AGIs alone (IC_025_/ROR_025_ = 0.132/1.097) (**Figures 4C, D**), while there was a decrease in signal strength for noninfectious myocarditis/pericarditis (SMQ) in combination therapy (IC_025_/ROR_025_ = 0.298/1.244) compared with ICIs alone (IC_025_/ROR_025_ = 0.702/1.630) (**Figures 4E, F**)

## Discussion

The synergistic antitumour effects of the combination therapy of ICIs and AGIs have been verified in emerging clinical studies, which displayed promising treatment efficacy in some solid tumours (2–9), such as atezolizumab plus bevacizumab for NSCLC (trial of IMpower150) (18) and HCC (Imbrave150) (19), avelumab plus axitinib for RCC (20), pembrolizumab plus axitinib for RCC (KEYNOTE-426) (21), pembrolizumab plus lenvatinib for RCC (CLEAR) (22) and endometrial cancer (KEYNOTE-775) (23), nivolumab plus cabozantinib for RCC (CheckMate 9ER) (24). Recently, nivolumab plus cabozantinib has been recommended as first-line therapy for advanced clear cell RCC by the European Society of Medical Oncology (ESMO) (25) and the FDA (9). However, in addition to its synergistic antitumour effects (efficacy), caution for the potential synergistic cardiovascular toxic effects (safety) of combination therapy is warranted, as both agents predispose patients to cardiovascular toxicity via different mechanisms. Currently, the cardiovascular safety profiles of combination therapy with ICIs and AGIs are unclear.

### ICIs combined with AGIs: increased overall cardiovascular risk

Firstly, since emerging combination approaches are under investigation, we found that the reported proportion of cardiovascular AEs for combination therapy with ICIs and AGIs increased yearly from 2014 to 2021, with a rapid increase since 2019. Secondly, disproportionality analysis demonstrated that cardiovascular AEs were overreported in patients using combination therapy, with stronger signal strength based on IC_025_ or ROR_025_ than in those using ICIs alone or AGIs alone as a whole. Thirdly, further analysis among indications of malignant tumours depicted similar findings for combination therapy. In summary, the present study showed that combination therapy with ICIs and AGIs may be associated with overreported cardiovascular AEs overall compared with ICIs alone or AGIs alone. To the best of our knowledge, this is the first pharmacovigilance analysis report on the cardiovascular toxicity of ICIs combined with AGIs in real-word settings. Similarly, a retrospective cohort study of 252 patients with lung cancer who received or did not receive ICIs demonstrated that previous or concomitant AGIs or ICIs use was associated with an increased risk for developing major adverse cardiovascular events (hazard ratio 2.15, 95% confidence interval 1.05 to 4.37, *P* = 0.04) (26).

### Variances in cardiovascular safety profiles of combination therapy

Although overall increased cardiovascular risks were observed for combination therapy, the present study revealed some specific variances in the cardiovascular safety profiles of combination therapy. Interestingly, compared to ICIs alone or AGIs alone as a class of agent, combination therapy mainly raised the risk of embolic and thrombotic events, whereas it decreased the risk of noninfectious myocarditis/pericarditis, based on specific PTs or SMQs. These findings were further confirmed by analysis among indications of malignant tumours. Moreover, with respect to outcomes of cardiovascular AEs, the present study revealed that combination therapy presented a lower frequency of death and life-threatening AEs than ICIs alone or AGIs alone overall. In particular, combination therapy showed a lower frequency of death and life-threatening noninfectious myocarditis/pericarditis (SMQ) than ICIs alone but a slightly higher frequency of death and life-threatening embolic and thrombotic events (SMQ) than AGIs alone. To the best of our knowledge, this is also the first report that has revealed such specific cardiovascular safety profiles of combination therapy.

We believe that these findings have great clinical implications. Noninfectious myocarditis is the most important cardiovascular AE for ICIs. Despite its low occurrence, the clinical course of ICI-associated myocarditis is often fulminant, and mortality rates up to 50% have been observed (27, 28), which is further confirmed by our present study (the frequency of death and life-threatening events was 49.2% in noninfectious myocarditis/pericarditis for ICIs alone). However, our study revealed that ICIs combined with AGIs can markedly decrease the risk of occurrence as well as the mortality of noninfectious myocarditis in comparison with ICIs alone. In short, it is suggested that combination therapy not only increased the antitumour effects but also decreased some severe cardiovascular AEs. Further studies to confirm our findings are highly warranted.

### Mechanisms of synergistic antitumour effects with varied cardiovascular risk

Notably, there is an intricate interplay between angiogenesis and immunity in tumours. In addition to its pivotal role in mediating tumour angiogenesis, a growing amount of evidence has revealed that VEGF also plays a critical role in the establishment of an immunosuppressive tumour microenvironment (TME) via several mechanisms (4, 6, 9, 13). Consequently, AGIs targeting VEGF can convert the TME from immunosuppressive to immunosupportive to enhance antitumour immunity. Therefore, combination therapy with ICIs and AGIs can demonstrate synergistic antitumour effects.

In contrast, more attention should be given to the potential increased risk of cardiovascular AEs associated with combination therapy. Firstly, hypertension seems to predispose patients to ICI-induced cardiovascular AEs, as shown in a retrospective study of 1 215 patients using ICIs (hazard ratio 3.19, *P* = 0.003) (29). Given that hypertension is the most reported cardiovascular AE for patients taking AGIs, the prohypertensive effect of AGIs may synergize with the cardiovascular toxicity of ICIs. Secondly, emerging evidence suggests that the application of ICIs is associated with more cardiovascular events mediated by the accelerated progression of atherosclerosis (30, 31), which is consistent with a previous study showing that VEGF inhibition disrupts endothelial homeostasis and accelerates atherosclerosis (32). Hence, ICIs and AGIs may increase atherosclerosis-related cardiovascular AEs. Finally, AGIs targeting VEGF can lead to endothelial cell damage that predisposes patients to embolic and thrombotic events (33). Furthermore, both venous and arterial thrombosis have been increasingly reported as cardiovascular AEs associated with ICIs in recent studies (34). Consequently, ICIs combined with AGIs can remarkably increase the risk of embolic and thrombotic events as demonstrated in our findings. Accordingly, the possible coping strategies for the cardiovascular AEs associated with combination therapy may be close follow-up, early detection and timely treatment with anticoagulant therapy for embolic and thrombotic events.

Although the mechanisms underlying ICI-induced myocarditis remain elusive to date, infiltration of active CD8+ T lymphocytes in the cardiac tissue and T-cell-induced autoimmunity have been implicated in the pathogenesis of ICI-associated myocarditis. Importantly, a previous clinical study revealed that atezolizumab plus bevacizumab can increase the number of intratumoral CD8+ T cells and tumour antigen-specific T-cell migration (35). Thus, it is speculated that ICIs and AGIs combination therapy can promote proper modulation of the immune TME to enhance antitumour immunity without increasing the risk of cardiac-specific autoimmunity and consequently decrease the risk of noninfectious myocarditis. Similarly, these speculations warrant future investigation.

Of note, Dr. Jain proposed the concept that an appropriate dose of antiangiogenic treatment can lead to normalization of the tumour vasculature, which can enhance cancer immunotherapy (36–38), while high-dose antiangiogenic treatments can promote tumour progression via multiple mechanisms (4). Although the effects of dose titration of AGIs on the immune TME remain unclear, future studies on combination therapy should take into account the appropriate dosing of AGIs and their timing or scheduling with ICIs so as to optimize the synergistic effects without significantly increasing cardiovascular risk.

We acknowledge that there are some limitations to be considered. Firstly, as a spontaneous reporting database with safety reports from various countries and different types of reporters, some biases may exist in the FAERS database due to the incompleteness of data reported. Secondly, due to the lack of baseline clinical characteristics or prior cardiovascular risk profiles, the FAERS database may suffer from reporting biases or confounding issues. Thirdly, the FAERS database does not contain data on the total number of patients treated with drugs, thus, the incidence of suspected drugs and AEs could not be calculated. Finally, a causal relationship cannot be confirmed in a retrospective study. Nevertheless, despite the intrinsic limitations mentioned above, the current study still provides some valuable clues of cardiovascular AE profiles for combination therapy that warrant further prospective studies.

## Conclusion

Overall, ICIs combined with AGIs were associated with overreported cardiovascular AEs, which mainly increased the risk of embolic and thrombotic events while decreasing the risk of noninfectious myocarditis/pericarditis compared to ICIs alone. Moreover, combination therapy demonstrated a lower frequency of death and life-threatening noninfectious myocarditis/pericarditis and embolic and thrombotic events than ICIs alone.

Of note, these findings on specific cardiovascular safety profiles may provide some valuable insights for the individual choice of combination therapy in clinical practice given that ICIs combined with AGIs have demonstrated promising synergistic antitumour effects and are likely to become essential treatments for multiple types of cancers. Above all, further studies to confirm appropriate dosing and scheduling of combination therapy to optimize the synergistic effect with reduced cardiovascular toxicity are highly warranted.

## Data availability statement

The original contributions presented in the study are included in the article/**Supplementary Material**. Further inquiries can be directed to the corresponding author.

## Ethics statement

Ethical review and approval was not required for the study on human participants in accordance with the local legislation and institutional requirements. Written informed consent from the participants’ legal guardian/next of kin was not required to participate in this study in accordance with the national legislation and the institutional requirements.

## Author contributions

YW was responsible for the study design. CC and LD were responsible for writing the article. XR was responsible for data acquisition and processing analysis. CC and LW were responsible for data checking and interpretation. All authors contributed to the article and approved the submitted version.
