# Supplementary material for: Cardiovascular toxicity profiles of immune checkpoint inhibitors with or without angiogenesis inhibitors: a real-world pharmacovigilance analysis based on the FAERS database from 2014 to 2022
Source: Front Immunol. 2023 May 24;14:1127128. doi: 10.3389/fimmu.2023.1127128 (PMC10244526; doi:10.3389/fimmu.2023.1127128)
Supplement: Supplementary file 1 [file DataSheet_1.pdf]

Table S1. SMQ “Cardiac arrhythmia” and PTs used according to MedDRA 24.0

Chronotropic incompetence	Paroxysmal atrioventricular block
Electrocardiogram repolarisation abnormality	Sinoatrial block
Electrocardiogram RR interval prolonged	Trifascicular block
Electrocardiogram U wave inversion	Ventricular dyssynchrony
Electrocardiogram U wave present	Wolff-Parkinson-White syndrome
Electrocardiogram U-wave abnormality	Nodal arrhythmia
Sudden cardiac death	Nodal rhythm
Bezold-Jarisch reflex	Sinus arrest
Bradycardia	Sinus arrhythmia
Cardiac arrest	Sinus bradycardia
Cardiac death	Sinus node dysfunction
Cardiac telemetry abnormal	Wandering pacemaker
Cardio-respiratory arrest	Arrhythmia
Central bradycardia	Heart alternation
Electrocardiogram abnormal	Heart rate irregular
Electrocardiogram ambulatory abnormal	Holiday heart syndrome
Electrocardiogram change	Pacemaker generated arrhythmia
Heart rate abnormal	Pacemaker syndrome
Heart rate decreased	Paroxysmal arrhythmia
Heart rate increased	Pulseless electrical activity
Loss of consciousness	Reperfusion arrhythmia
Palpitations	Withdrawal arrhythmia
Rebound tachycardia	Arrhythmia supraventricular
Respiratory sinus arrhythmia magnitude abnormal	Atrial fibrillation
Respiratory sinus arrhythmia magnitude decreased	Atrial flutter
Respiratory sinus arrhythmia magnitude increased	Atrial parasystole
Sudden death	Atrial tachycardia
Syncope	Junctional ectopic tachycardia
Tachycardia	Sinus tachycardia
Tachycardia paroxysmal	Supraventricular extrasystoles
Bradyarrhythmia	Supraventricular tachyarrhythmia
Ventricular asystole	Supraventricular tachycardia
Accessory cardiac pathway	ECG P wave inverted
Adams-Stokes syndrome	Electrocardiogram P wave abnormal
Agonal rhythm	Retrograde p-waves
Atrial conduction time prolongation	Anomalous atrioventricular excitation
Atrioventricular block	Cardiac fibrillation
Atrioventricular block complete	Cardiac flutter
Atrioventricular block first degree	Extrasystoles
Atrioventricular block second degree	Tachyarrhythmia
Atrioventricular conduction time shortened	Accelerated idioventricular rhythm

Atrioventricular dissociation	Cardiac fibrillation
Atrioventricular node dysfunction	Parasystole
Bifascicular block	Rhythm idioventricular
BRASH syndrome	Torsade de pointes
Brugada syndrome	Ventricular arrhythmia
Bundle branch block	Ventricular extrasystoles
Bundle branch block bilateral	Ventricular fibrillation
Bundle branch block left	Ventricular flutter
Bundle branch block right	Ventricular parasystole
Conduction disorder	Ventricular pre-excitation
Defect conduction intraventricular	Ventricular tachyarrhythmia
Electrocardiogram delta waves abnormal	Ventricular tachycardia
Electrocardiogram PR prolongation	Andersen-Tawil syndrome
Electrocardiogram PR shortened	Arrhythmogenic right ventricular dysplasia
Electrocardiogram QRS complex prolonged	Atrioventricular node dispersion
Electrocardiogram QT prolonged	Brugada syndrome
Electrocardiogram repolarisation abnormality	Junctional ectopic tachycardia
Lenegre's disease	Lown-Ganong-Levine syndrome

Table S2. SMQ "Cardiac failure" and PTs used according to MedDRA 24.0

Acute left ventricular failure	Cardiothoracic ratio increased
Acute pulmonary oedema	Central venous pressure increased
Acute right ventricular failure	Coronary sinus dilatation
Cardiac asthma	Diastolic dysfunction
Cardiac failure	Dilatation ventricular
Cardiac failure acute	Dyspnoea paroxysmal nocturnal
Cardiac failure chronic	Heart transplant
Cardiac failure congestive	Hepatic vein dilatation
Cardiac failure high output	Implantable cardiac monitor replacement
Cardiogenic shock	Intracardiac pressure increased
Cardiohepatic syndrome	Jugular vein distension
Cardiopulmonary failure	Left ventricular diastolic collapse
Cardiorenal syndrome	Left ventricular dilatation
Chronic left ventricular failure	Left ventricular dysfunction
Chronic right ventricular failure	Left ventricular enlargement
Congestive hepatopathy	Lower respiratory tract congestion
Cor pulmonale	Myocardial depression
Cor pulmonale acute	Nocturnal dyspnoea
Cor pulmonale chronic	N-terminal prohormone brain natriuretic peptide abnormal
Ejection fraction decreased	N-terminal prohormone brain natriuretic peptide increased
Hepatojugular reflux	Oedema
Left ventricular failure	Oedema blister
Low cardiac output syndrome	Oedema due to cardiac disease
Obstructive shock	Oedema peripheral
Pulmonary oedema	Orthopnoea
Radiation associated cardiac failure	Peripheral swelling
Right ventricular ejection fraction decreased	Post cardiac arrest syndrome
Right ventricular failure	Prohormone brain natriuretic peptide abnormal
Ventricular failure	Prohormone brain natriuretic peptide increased
Artificial heart implant	Pulmonary congestion
Atrial natriuretic peptide abnormal	Right ventricular diastolic collapse
Atrial natriuretic peptide increased	Right ventricular dilatation
Bendopnoea	Right ventricular dysfunction
Brain natriuretic peptide abnormal	Right ventricular enlargement
Brain natriuretic peptide increased	Scan myocardial perfusion abnormal
Cardiac cirrhosis	Stroke volume decreased
Cardiac contractility modulation therapy	Surgical ventricular restoration
Cardiac device reprogramming	Systolic dysfunction
Cardiac dysfunction	Temporary mechanical circulatory support
Cardiac index decreased	Venous pressure increased
Cardiac output decreased	Venous pressure jugular abnormal

Cardiac resynchronisation therapy	Venous pressure jugular increased
Cardiac ventriculogram abnormal	Ventricular assist device insertion
Cardiac ventriculogram left abnormal	Ventricular compliance decreased
Cardiac ventriculogram right abnormal	Ventricular dysfunction
Cardiomegaly	Ventricular dyssynchrony
Cardio-respiratory distress	Wall motion score index abnormal

Table S3. SMQ "Cardiomyopathy" and PTs used according to MedDRA 24.0

Atrial septal defect acquired	Computerised tomogram thorax abnormal
Biopsy heart abnormal	Decreased ventricular preload
Cardiac amyloidosis	Diastolic dysfunction
Cardiac hypertrophy	Dilatation atrial
Cardiac iron overload	Dilatation ventricular
Cardiac sarcoidosis	Directional Doppler flow tests abnormal
Cardiac septal hypertrophy	Dynamic cardiomyoplasty
Cardiomyopathy	Dyspnoea
Cardiomyopathy acute	ECG signs of ventricular hypertrophy
Cardiomyopathy alcoholic	Echocardiogram abnormal
Cardiotoxicity	Electrocardiogram abnormal
Chagas' cardiomyopathy	Electrocardiogram change
Congestive cardiomyopathy	Electrocardiogram PR segment depression
Diabetic cardiomyopathy	Electrocardiogram U wave inversion
Ejection fraction abnormal	Endocardial fibroelastosis
Ejection fraction decreased	External counterpulsation
Eosinophilic myocarditis	Heart and lung transplant
Giant cell myocarditis	Heart transplant
Hypertensive cardiomyopathy	Heart transplant failure
Hypertrophic cardiomyopathy	Heart-lung transplant failure
Ischaemic cardiomyopathy	Hepatomegaly
Metabolic cardiomyopathy	Hyperdynamic left ventricle
Mitochondrial cardiomyopathy	Hypersensitivity myocarditis
Myocardial calcification	Immune-mediated myocarditis
Myocardial fibrosis	Implantable cardiac monitor replacement
Myocardial haemorrhage	Increased ventricular preload
Non-obstructive cardiomyopathy	Intracardiac pressure increased
Obesity cardiomyopathy	Irregular breathing
Pacing induced cardiomyopathy	Labile blood pressure
Peripartum cardiomyopathy	Left atrial dilatation
Pulmonary arterial wedge pressure increased	Left atrial enlargement
Restrictive cardiomyopathy	Left atrial volume abnormal
Right ventricular ejection fraction decreased	Left atrial volume decreased
Stress cardiomyopathy	Left atrial volume increased
Tachycardia induced cardiomyopathy	Left ventricle outflow tract obstruction
Thyrotoxic cardiomyopathy	Left ventricular dilatation
Toxic cardiomyopathy	Left ventricular dysfunction
Ventricular septal defect acquired	Left ventricular end-diastolic pressure decreased
Abnormal precordial movement	Left ventricular enlargement
Acquired cardiac septal defect	Left ventricular failure
Acute left ventricular failure	Left ventricular heave

Alcohol septal ablation	Lung opacity
Arrhythmia	Lupus myocarditis
Arrhythmia supraventricular	Magnetic resonance imaging thoracic abnormal
Arrhythmic storm	Malarial myocarditis
Artificial heart implant	Mental status changes
Ascites	Multiple gated acquisition scan abnormal
Atrial enlargement	Myocardiac abscess
Atrial hypertrophy	Myocardial injury
Atrial pressure increased	Myocardial necrosis marker increased
Autoimmune myocarditis	Myocardial strain imaging abnormal
Bendopnoea	Myocarditis
Blood pressure diastolic abnormal	Myoglobinaemia
Blood pressure diastolic decreased	Myoglobinuria
Blood pressure diastolic increased	Myopericarditis
Blood pressure fluctuation	Nocturia
Blood pressure inadequately controlled	Oedema
Blood pressure systolic abnormal	Orthostatic hypotension
Blood pressure systolic decreased	Palpitations
Blood pressure systolic increased	Papillary muscle disorder
Cardiac aneurysm	Papillary muscle haemorrhage
Cardiac arrest	Radiation myocarditis
Cardiac contractility decreased	Right atrial dilatation
Cardiac contractility modulation therapy	Right atrial enlargement
Cardiac device reprogramming	Right atrial pressure increased
Cardiac dysfunction	Right ventricle outflow tract obstruction
Cardiac electrophysiologic study abnormal	Right ventricular dilatation
Cardiac failure	Right ventricular enlargement
Cardiac failure acute	Right ventricular heave
Cardiac failure chronic	Right ventricular systolic pressure decreased
Cardiac failure congestive	Scan myocardial perfusion abnormal
Cardiac function test abnormal	Sudden cardiac death
Cardiac imaging procedure abnormal	Sudden death
Cardiac index abnormal	Surgical ventricular restoration
Cardiac index decreased	Syncope
Cardiac index increased	Systolic anterior motion of mitral valve
Cardiac monitoring abnormal	Systolic dysfunction
Cardiac operation	Temporary mechanical circulatory support
Cardiac output decreased	Ultrasound Doppler abnormal
Cardiac pseudoaneurysm	Vascular resistance pulmonary increased
Cardiac resynchronisation therapy	Ventricular arrhythmia
Cardiac ventricular scarring	Ventricular assist device insertion
Cardiac ventriculogram abnormal	Ventricular dysfunction
Cardiac ventriculogram left abnormal	Ventricular dyskinesia

Cardiac ventriculogram right abnormal	Ventricular dyssynchrony
Cardiomegaly	Ventricular enlargement
Cardiothoracic ratio increased	Ventricular hyperkinesia
Cardiovascular disorder	Ventricular hypertrophy
Cardiovascular function test abnormal	Ventricular hypokinesia
Chest pain	Ventricular remodelling
Chest X-ray abnormal	Wall motion score index abnormal

Table S4. SMQ "Embolic and thrombotic events" and PTs used according to MedDRA 24.0

Acute aortic syndrome	Pulmonary microemboli
Acute coronary syndrome	Pulmonary thrombosis
Acute myocardial infarction	Pulmonary vein occlusion
Amaurosis	Pulmonary veno-occlusive disease
Amaurosis fugax	Pulmonary venous thrombosis
Aneurysm thrombosis	Renal vein embolism
Angioplasty	Renal vein occlusion
Aortic bypass	Renal vein thrombosis
Aortic embolus	Retinal vein occlusion
Aortic surgery	Retinal vein thrombosis
Aortic thrombosis	SI QIII TIII pattern
Aortogram abnormal	Sigmoid sinus thrombosis
Arterectomy	Splenic vein occlusion
Arterectomy with graft replacement	Splenic vein thrombosis
Arterial angioplasty	Subclavian vein occlusion
Arterial bypass operation	Subclavian vein thrombosis
Arterial graft	Superficial vein thrombosis
Arterial occlusive disease	Superior sagittal sinus thrombosis
Arterial revascularisation	Superior vena cava occlusion
Arterial stent insertion	Superior vena cava syndrome
Arterial therapeutic procedure	Thrombophlebitis
Arterial thrombosis	Thrombophlebitis migrans
Arteriogram abnormal	Thrombosed varicose vein
Arteriogram carotid abnormal	Thrombosis corpora cavernosa
Arteriotomy	Transverse sinus thrombosis
Atherectomy	Vena cava embolism
Atherosclerotic plaque rupture	Vena cava filter insertion
Atrial appendage closure	Vena cava filter removal
Atrial appendage resection	Vena cava thrombosis
Basal ganglia infarction	Venogram abnormal
Basilar artery occlusion	Venoocclusive disease
Basilar artery thrombosis	Venoocclusive liver disease
Blindness transient	Venous angioplasty
Brachiocephalic artery occlusion	Venous occlusion
Capsular warning syndrome	Venous operation
Carotid angioplasty	Venous recanalisation
Carotid arterial embolus	Venous repair
Carotid artery bypass	Venous stent insertion
Carotid artery occlusion	Venous thrombosis
Carotid artery stent insertion	Venous thrombosis in pregnancy
Carotid artery thrombosis	Venous thrombosis limb

Carotid endarterectomy	Visceral venous thrombosis
Cerebellar artery occlusion	Administration site thrombosis
Cerebellar artery thrombosis	Adrenal thrombosis
Cerebral artery embolism	Angiogram abnormal
Cerebral artery occlusion	Angiogram cerebral abnormal
Cerebral artery stent insertion	Angiogram peripheral abnormal
Cerebral artery thrombosis	Antiphospholipid syndrome
Cerebral hypoperfusion	Application site thrombosis
Cerebrovascular insufficiency	Arteriovenous fistula occlusion
Cerebrovascular stenosis	Arteriovenous fistula thrombosis
Coeliac artery occlusion	Arteriovenous graft thrombosis
Coronary angioplasty	Artificial blood vessel occlusion
Coronary arterial stent insertion	Atrial thrombosis
Coronary artery bypass	Autoimmune heparin-induced thrombocytopenia
Coronary artery embolism	Basal ganglia stroke
Coronary artery occlusion	Bone infarction
Coronary artery reocclusion	Brain stem embolism
Coronary artery surgery	Brain stem infarction
Coronary artery thrombosis	Brain stem stroke
Coronary endarterectomy	Brain stem thrombosis
Coronary revascularisation	Cardiac ventricular thrombosis
Coronary vascular graft occlusion	Catheter directed thrombolysis
Embolia cutis medicamentosa	Catheter site thrombosis
Embolism arterial	Cerebellar embolism
Endarterectomy	Cerebellar infarction
Femoral artery embolism	Cerebral congestion
Hepatic artery embolism	Cerebral infarction
Hepatic artery occlusion	Cerebral ischaemia
Hepatic artery thrombosis	Cerebral microembolism
Hypothenar hammer syndrome	Cerebral microinfarction
Iliac artery embolism	Cerebral thrombosis
Iliac artery occlusion	Cerebral vascular occlusion
Internal capsule infarction	Cerebrospinal thrombotic tamponade
Intra-aortic balloon placement	Cerebrovascular accident
Intraoperative cerebral artery occlusion	Cerebrovascular accident prophylaxis
Ischaemic cerebral infarction	Cerebrovascular disorder
Ischaemic stroke	Cerebrovascular operation
Lacunar infarction	Choroidal infarction
Left atrial appendage closure implant	Collateral circulation
Leriche syndrome	Coronary bypass thrombosis
Mesenteric arterial occlusion	Device embolisation
Mesenteric arteriosclerosis	Device occlusion
Mesenteric artery embolism	Device related thrombosis

Mesenteric artery stenosis	Diplegia
Mesenteric artery stent insertion	Directional Doppler flow tests abnormal
Mesenteric artery thrombosis	Disseminated intravascular coagulation
Metabolic stroke	Disseminated intravascular coagulation in newborn
Myocardial infarction	Embolic cerebellar infarction
Myocardial necrosis	Embolic cerebral infarction
Ophthalmic artery occlusion	Embolic pneumonia
Ophthalmic artery thrombosis	Embolic stroke
Papillary muscle infarction	Embolism
Penile artery occlusion	Eye infarction
Percutaneous coronary intervention	Fluorescence angiogram abnormal
Peripheral arterial occlusive disease	Gastric infarction
Peripheral arterial reocclusion	Graft thrombosis
Peripheral artery angioplasty	Haemorrhagic adrenal infarction
Peripheral artery bypass	Haemorrhagic cerebral infarction
Peripheral artery occlusion	Haemorrhagic infarction
Peripheral artery stent insertion	Haemorrhagic stroke
Peripheral artery surgery	Haemorrhagic transformation stroke
Peripheral artery thrombosis	Haemorrhoids thrombosed
Peripheral embolism	Hemiparesis
Peripheral endarterectomy	Hemiplegia
Popliteal artery entrapment syndrome	Heparin-induced thrombocytopenia
Post procedural myocardial infarction	Hepatic infarction
Postinfarction angina	Hepatic vascular thrombosis
Precerebral artery embolism	Implant site thrombosis
Precerebral artery occlusion	Incision site vessel occlusion
Precerebral artery thrombosis	Infarction
Profundaplasty	Infusion site thrombosis
Pseudo-occlusion of internal carotid artery	Injection site thrombosis
Pulmonary artery occlusion	Inner ear infarction
Pulmonary artery therapeutic procedure	Instillation site thrombosis
Pulmonary artery thrombosis	Intestinal infarction
Pulmonary endarterectomy	Intracardiac mass
Pulmonary tumour thrombotic microangiopathy	Intracardiac thrombus
Renal artery angioplasty	Lambl's excrescences
Renal artery occlusion	Medical device site thrombosis
Renal artery thrombosis	Mesenteric vascular insufficiency
Renal embolism	Mesenteric vascular occlusion
Renal-limited thrombotic microangiopathy	Microembolism
Retinal artery embolism	Monoparesis
Retinal artery occlusion	Monoplegia
Retinal artery thrombosis	Muscle infarction
Segmental arterial mediolysis	Optic nerve infarction

Silent myocardial infarction	Pancreatic infarction
Spinal artery embolism	Paradoxical embolism
Spinal artery thrombosis	Paraneoplastic thrombosis
Splenic artery thrombosis	Paraparesis
Splenic embolism	Paraplegia
Stress cardiomyopathy	Paresis
Subclavian artery embolism	Peripheral revascularisation
Subclavian artery occlusion	Pituitary infarction
Subclavian artery thrombosis	Placental infarction
Thromboembolectomy	Pneumatic compression therapy
Thrombotic microangiopathy	Portal shunt procedure
Thrombotic thrombocytopenic purpura	Post procedural stroke
Transient ischaemic attack	Postpartum thrombosis
Truncus coeliacus thrombosis	Prosthetic cardiac valve thrombosis
Vascular pseudoaneurysm thrombosis	Prosthetic vessel implantation
Vertebral artery occlusion	Quadriparesis
Vertebral artery thrombosis	Quadriplegia
Visual acuity reduced transiently	Renal infarct
Aseptic cavernous sinus thrombosis	Renal vascular thrombosis
Axillary vein thrombosis	Retinal infarction
Brachiocephalic vein occlusion	Retinal vascular thrombosis
Brachiocephalic vein thrombosis	Revascularisation procedure
Budd-Chiari syndrome	Shunt occlusion
Catheterisation venous	Shunt thrombosis
Cavernous sinus thrombosis	Spinal cord infarction
Central venous catheterisation	Spinal stroke
Cerebral venous sinus thrombosis	Splenic infarction
Cerebral venous thrombosis	Splenic thrombosis
Compression garment application	Spontaneous heparin-induced thrombocytopenia syndrome
Deep vein thrombosis	Stoma site thrombosis
Deep vein thrombosis postoperative	Stroke in evolution
Embolism venous	Strokectomy
Hepatic vein embolism	Surgical vascular shunt
Hepatic vein occlusion	Testicular infarction
Hepatic vein thrombosis	Thalamic infarction
Homans' sign positive	Thrombectomy
Iliac vein occlusion	Thromboangiitis obliterans
Inferior vena cava syndrome	Thrombolysis
Inferior vena caval occlusion	Thrombosis
Jugular vein embolism	Thrombosis in device
Jugular vein occlusion	Thrombosis mesenteric vessel
Jugular vein thrombosis	Thrombosis prophylaxis
Mahler sign	Thrombosis with thrombocytopenia syndrome

May-Thurner syndrome	Thrombotic cerebral infarction
Mesenteric vein thrombosis	Thrombotic stroke
Mesenteric venous occlusion	Thyroid infarction
Obstetrical pulmonary embolism	Tumour embolism
Obstructive shock	Tumour thrombectomy
Ophthalmic vein thrombosis	Tumour thrombosis
Ovarian vein thrombosis	Ultrasonic angiogram abnormal
Paget-Schroetter syndrome	Ultrasound Doppler abnormal
Pelvic venous thrombosis	Umbilical cord occlusion
Penile vein thrombosis	Umbilical cord thrombosis
Peripheral vein occlusion	Vaccination site thrombosis
Peripheral vein thrombosis	Vascular access site thrombosis
Peripheral vein thrombus extension	Vascular device occlusion
Phlebectomy	Vascular graft
Portal vein cavernous transformation	Vascular graft occlusion
Portal vein embolism	Vascular graft thrombosis
Portal vein occlusion	Vascular operation
Portal vein thrombosis	Vascular stent insertion
Portosplenomesenteric venous thrombosis	Vascular stent occlusion
Post procedural pulmonary embolism	Vascular stent thrombosis
Post thrombotic syndrome	Vasodilation procedure
Postoperative thrombosis	Vessel puncture site occlusion
Postpartum venous thrombosis	Vessel puncture site thrombosis
Pulmonary embolism	Visual midline shift syndrome
Pulmonary infarction	

Table S5. SMQ “Hypertension” and PTs used according to MedDRA 24.0

Accelerated hypertension	Retinopathy hypertensive
Blood pressure ambulatory increased	Secondary aldosteronism
Blood pressure diastolic increased	Secondary hypertension
Blood pressure inadequately controlled	Superimposed pre-eclampsia
Blood pressure increased	Supine hypertension
Blood pressure management	Systolic hypertension
Blood pressure orthostatic increased	Withdrawal hypertension
Blood pressure systolic increased	Aldosterone urine abnormal
Catecholamine crisis	Aldosterone urine increased
Dialysis induced hypertension	Angiotensin converting enzyme abnormal
Diastolic hypertension	Angiotensin converting enzyme increased
Eclampsia	Angiotensin I abnormal
Endocrine hypertension	Angiotensin I increased
Essential hypertension	Angiotensin II abnormal
Gestational hypertension	Angiotensin II increased
HELLP syndrome	Angiotensin II receptor type 1 antibody positive
Hyperaldosteronism	Blood aldosterone abnormal
Hypertension	Blood aldosterone increased
Hypertensive angiopathy	Blood catecholamines abnormal
Hypertensive cardiomegaly	Blood catecholamines increased
Hypertensive cardiomyopathy	Blood pressure abnormal
Hypertensive cerebrovascular disease	Blood pressure ambulatory abnormal
Hypertensive crisis	Blood pressure diastolic abnormal
Hypertensive emergency	Blood pressure fluctuation
Hypertensive encephalopathy	Blood pressure orthostatic abnormal
Hypertensive end-organ damage	Blood pressure systolic abnormal
Hypertensive heart disease	Catecholamines urine abnormal
Hypertensive nephropathy	Catecholamines urine increased
Hypertensive urgency	Diuretic therapy
Labile hypertension	Ectopic aldosterone secretion
Malignant hypertension	Ectopic renin secretion
Malignant hypertensive heart disease	Epinephrine abnormal
Malignant renal hypertension	Epinephrine increased
Maternal hypertension affecting foetus	Labile blood pressure
Mean arterial pressure increased	Metanephrine urine abnormal
Metabolic syndrome	Metanephrine urine increased
Neurogenic hypertension	Non-dipping
Orthostatic hypertension	Norepinephrine abnormal
Page kidney	Norepinephrine increased
Postoperative hypertension	Normetanephrine urine increased
Pre-eclampsia	Pseudoaldosteronism

Prehypertension	Renal vascular resistance increased
Procedural hypertension	Renin abnormal
Renal hypertension	Renin increased
Renal sympathetic nerve ablation	Renin-angiotensin system inhibition
Renovascular hypertension	Tyramine reaction

Table S6. SMQ "Ischaemic heart disease" and PTs used according to MedDRA 24.0

Acute cardiac event	Coronary artery compression
Acute coronary syndrome	Coronary artery disease
Acute myocardial infarction	Coronary artery dissection
Angina unstable	Coronary artery insufficiency
Blood creatine phosphokinase MB abnormal	Coronary artery restenosis
Blood creatine phosphokinase MB increased	Coronary artery stenosis
Coronary artery embolism	Coronary artery surgery
Coronary artery occlusion	Coronary brachytherapy
Coronary artery reocclusion	Coronary bypass stenosis
Coronary artery thrombosis	Coronary endarterectomy
Coronary bypass thrombosis	Coronary no-reflow phenomenon
Coronary vascular graft occlusion	Coronary ostial stenosis
Kounis syndrome	Coronary revascularisation
Myocardial infarction	Coronary steal syndrome
Myocardial necrosis	Coronary vascular graft stenosis
Myocardial reperfusion injury	Diabetic coronary microangiopathy
Myocardial stunning	ECG signs of myocardial ischaemia
Papillary muscle infarction	External counterpulsation
Periprocedural myocardial infarction	Haemorrhage coronary artery
Post procedural myocardial infarction	Ischaemic cardiomyopathy
Postinfarction angina	Ischaemic mitral regurgitation
Silent myocardial infarction	Microvascular coronary artery disease
Troponin I increased	Myocardial hypoperfusion
Troponin increased	Myocardial hypoxia
Troponin T increased	Myocardial ischaemia
Blood creatine phosphokinase abnormal	Percutaneous coronary intervention
Blood creatine phosphokinase increased	Prinzmetal angina
Cardiac ventricular scarring	Stress cardiomyopathy
ECG electrically inactive area	Subclavian coronary steal syndrome
ECG signs of myocardial infarction	Subendocardial ischaemia
Electrocardiogram Q wave abnormal	Wellens' syndrome
Electrocardiogram ST segment abnormal	Arterial revascularisation
Electrocardiogram ST segment elevation	Arteriogram coronary abnormal
Electrocardiogram ST-T segment elevation	Cardiac stress test abnormal
Electrocardiogram U wave inversion	Cardiopulmonary exercise test abnormal
Infarction	Cardiovascular event prophylaxis
Myocardial necrosis marker increased	Computerised tomogram coronary artery abnormal
Scan myocardial perfusion abnormal	Elastic vessel recoil complication
Vascular graft occlusion	Electrocardiogram PR segment depression
Vascular stent occlusion	Electrocardiogram ST segment depression
Vascular stent thrombosis	Electrocardiogram ST-T segment abnormal

Acute cardiac event	Electrocardiogram ST-T segment depression
Angina pectoris	Electrocardiogram T wave abnormal
Angina unstable	Electrocardiogram T wave inversion
Anginal equivalent	Electrocardiogram U wave inversion
Arteriosclerosis coronary artery	Exercise electrocardiogram abnormal
Arteriospasm coronary	Exercise test abnormal
Cardiac perfusion defect	Post angioplasty restenosis
Chronic coronary syndrome	Restenosis
Coronary angioplasty	Stress echocardiogram abnormal
Coronary arterial stent insertion	Vascular stent stenosis
Coronary artery bypass	Wall motion score index abnormal

Table S7. SMQ "Noninfectious myocarditis/pericarditis" and PTs used according to MedDRA 24.0

Autoimmune myocarditis	Electrocardiogram ST-T change
Autoimmune pericarditis	Electrocardiogram ST-T segment abnormal
Carditis	Electrocardiogram ST-T segment depression
Chronic myocarditis	Electrocardiogram T wave abnormal
Eosinophilic myocarditis	Electrocardiogram T wave amplitude decreased
Giant cell myocarditis	Electrocardiogram T wave amplitude increased
Hypersensitivity myocarditis	Electrocardiogram T wave biphasic
Immune-mediated myocarditis	Electrocardiogram T wave inversion
Myocarditis	Exercise electrocardiogram abnormal
Myopericarditis	Extrasystoles
Pericarditis	Fascicular block
Pericarditis adhesive	Intracardiac thrombus
Pericarditis constrictive	Intrapericardial thrombosis
Pleuropericarditis	Left ventricular dilatation
Acute cardiac event	Left ventricular dysfunction
Acute left ventricular failure	Left ventricular enlargement
Acute right ventricular failure	Left ventricular failure
Arrhythmia supraventricular	Low cardiac output syndrome
Atrial conduction time prolongation	Lupus myocarditis
Atrial standstill	Myocardial calcification
Atrioventricular block	Myocardial injury
Atrioventricular block complete	Myocardial necrosis
Atrioventricular block first degree	Myocardial necrosis marker increased
Atrioventricular block second degree	Myocardial oedema
Atrioventricular conduction time shortened	Myocardial rupture
Atrioventricular dissociation	Myocardial strain imaging abnormal
Atrioventricular node dysfunction	Myosin increased
Bifascicular block	Paroxysmal arrhythmia
Biopsy heart abnormal	Pericardial disease
Biopsy pericardium abnormal	Pericardial drainage
Blood creatine phosphokinase MB increased	Pericardial drainage test abnormal
Bundle branch block	Pericardial effusion
Bundle branch block bilateral	Pericardial excision
Bundle branch block left	Pericardial haemorrhage
Bundle branch block right	Pericardial operation
Cardiac arrest	Pericardial repair
Cardiac assistance device user	Pericardial rub
Cardiac contractility decreased	Pericardiotomy
Cardiac contractility modulation therapy	Postpericardiotomy syndrome
Cardiac death	QRS axis abnormal

Cardiac failure	Red blood cell sedimentation rate increased
Cardiac failure acute	Right ventricular dilatation
Cardiac index decreased	Right ventricular dysfunction
Cardiac output decreased	Right ventricular ejection fraction decreased
Cardiac tamponade	Right ventricular enlargement
Cardiac ventriculogram abnormal	Right ventricular failure
Cardiac ventriculogram left abnormal	Scan myocardial perfusion abnormal
Cardiac ventriculogram right abnormal	Sinoatrial block
Cardiogenic shock	Sinus arrest
Cardiopulmonary failure	Sudden cardiac death
Cardio-respiratory arrest	Sudden death
Cardio-respiratory distress	Supraventricular extrasystoles
Cardiothoracic ratio increased	Supraventricular tachyarrhythmia
Defect conduction intraventricular	Supraventricular tachycardia
Dilatation ventricular	Tachyarrhythmia
Echocardiogram abnormal	Tachycardia paroxysmal
Ejection fraction decreased	Trifascicular block
Electrocardiogram abnormal	Troponin I increased
Electrocardiogram ambulatory abnormal	Troponin increased
Electrocardiogram change	Troponin T increased
Electrocardiogram low voltage	Ventricular compliance decreased
Electrocardiogram PR segment depression	Ventricular enlargement
Electrocardiogram QRS complex abnormal	Ventricular extrasystoles
Electrocardiogram QT interval abnormal	Ventricular failure
Electrocardiogram QT prolonged	Ventricular tachyarrhythmia
Electrocardiogram ST segment abnormal	Ventricular tachycardia
Electrocardiogram ST segment depression	Wall motion score index abnormal

Table S8. SMQ "Pulmonary hypertension" and PTs used according to MedDRA 24.0

Acute right ventricular failure	Atrial pressure increased
Cardiac ventriculogram right abnormal	Banti's syndrome
Central venous pressure increased	Bendopnoea
Chronic right ventricular failure	Brain natriuretic peptide increased
Cor pulmonale	Cardiac cirrhosis
Cor pulmonale acute	Cardiac index decreased
Cor pulmonale chronic	Cardiac murmur
Coronary sinus dilatation	Carotid pulse decreased
Portopulmonary hypertension	Combined pulmonary fibrosis and emphysema
Pulmonary arterial hypertension	Cyanosis central
Pulmonary arterial pressure abnormal	Diastolic dysfunction
Pulmonary arterial pressure increased	Dilatation ventricular
Pulmonary arterial wedge pressure increased	Dizziness exertional
Pulmonary artery dilatation	Dyspnoea
Pulmonary artery wall hypertrophy	Dyspnoea at rest
Pulmonary capillary haemangiomatosis	Dyspnoea exertional
Pulmonary endarterectomy	Dyspnoea paroxysmal nocturnal
Pulmonary hypertension	Exercise test abnormal
Pulmonary hypertensive crisis	Heart sounds abnormal
Pulmonary tumour thrombotic microangiopathy	Hepatojugular reflux
Pulmonary valve incompetence	Hypoxia
Pulmonary vascular resistance abnormality	Increased ventricular afterload
Pulmonary vein occlusion	Increased ventricular preload
Pulmonary vein stenosis	Intracardiac pressure increased
Pulmonary veno-occlusive disease	Irregular breathing
Pulmonary venous hypertension	Jugular vein distension
Right atrial dilatation	Left ventricular end-diastolic pressure decreased
Right atrial enlargement	Oedema due to cardiac disease
Right atrial hypertrophy	Orthopnoea
Right atrial pressure increased	Pulmonary arterial wedge pressure abnormal
Right atrial volume increased	Pulmonary arteriopathy
Right ventricular dilatation	Pulmonary artery arteriosclerosis
Right ventricular dysfunction	Pulmonary artery occlusion
Right ventricular ejection fraction decreased	Pulmonary vascular disorder
Right ventricular enlargement	Respiratory fatigue
Right ventricular failure	Stroke volume decreased
Right ventricular heave	Supra-aortic trunk stenosis
Right ventricular hypertension	Tricuspid valve prolapse
Right ventricular hypertrophy	Tricuspid valve repair
Right ventricular systolic pressure increased	Tricuspid valve replacement
Tricuspid valve incompetence	Tricuspid valve sclerosis

Vascular resistance pulmonary increased	Tricuspid valve thickening
Abnormal precordial movement	Venous pressure jugular increased
Angiogram pulmonary abnormal	Ventricular enlargement
Atrial enlargement	

Table S9. SMQ “Torsade de pointes/QT prolongation” and PTs used according to MedDRA 24.0

Electrocardiogram QT interval abnormal	Electrocardiogram U wave present
Electrocardiogram QT prolonged	Electrocardiogram U-wave abnormality
Long QT syndrome	Loss of consciousness
Torsade de pointes	Sudden cardiac death
Ventricular tachycardia	Sudden death
Arrhythmic storm	Syncope
Cardiac arrest	Ventricular arrhythmia
Cardiac death	Ventricular fibrillation
Cardiac fibrillation	Ventricular flutter
Cardio-respiratory arrest	Ventricular tachyarrhythmia
Electrocardiogram repolarisation abnormality	Electrocardiogram U wave present
Electrocardiogram U wave inversion	
