# Supplementary material for: Cardiovascular toxicity profiles of immune checkpoint inhibitors with or without angiogenesis inhibitors: a real-world pharmacovigilance analysis based on the FAERS database from 2014 to 2022
Source: Front Immunol. 2023 May 24;14:1127128. doi: 10.3389/fimmu.2023.1127128 (PMC10244526; doi:10.3389/fimmu.2023.1127128)
Supplement: Supplementary file 2 [file DataSheet_2.pdf]

Table S10. Thorough drug name archive of immune checkpoint inhibitor and angiogenesis inhibitor

Classes	Generic names	Brand names	Research codes
**Immune checkpoint inhibitor (ICI)**			
**Programmed death-1 (PD-1) inhibitor**	Nivolumab	Opdivo	BMS-936558; ONO-4538; DX-1106
	Pembrolizumab	Keytruda	MK-3475
	Cemiplimab	Libtayo	REGN2810; SAR439684
	Tislelizumab (China)		BGB-A317
	Toripalimab (China)		1924598-82-2
	Sintilimab (China)	Tyvyt	IBI-308
	Camrelizumab (China)		SHR-1210
	Penpulimab (China)		AK105
	Zimberelimab (China)		AB122
**Programmed death ligand-1 (PD-L1) inhibitor**	Atezolizumab	Tecentriq	MPDL3280A
	Durvalumab	Imfinzi	MEDI 4736
	Avelumab	Bavencio	MSB0010718C
	Envafolimab (China)		KN035
	Sugemalimab (China)	Cejemly	CS1001
**Cytotoxic T-lymphocyte antigen-4 (CTLA-4) inhibitor**	Ipilimumab	Yervoy	BMS-734016; MDX-010
	Tremelimumab		CP 675206
**Angiogenesis inhibitor (AGI)**			
**Anti-VEGF monoclonal antibody (mAb)**	Bevacizumab	Avastin	
	Bevacizumab-Awwb	Mvasi	
	Bevacizumab-Bvzr	Zirabev	PF-06439535
	Bevacizumab	Avastin	
		BYVASDA	
	Bevacizumab (China)	Pusintin	
		POBEVCY	
**Anti-VEGFR mAb**	Ramucirumab	Cyramza	IMC-1121B

**VEGF-trap**	Aflibercept	Zaltrap	BAY-865321
	Sorafenib	Nexavar	BAY 43-9006
	Sunitinib	Sutent	SU11248
	Pazopanib	Votrient	GW786034
	Vandetanib	Caprelsa	ZD6474
	Cabozantinib	Cabometyx	XL184
	Regorafenib	Stivarga	BAY 73-4506
	Axitinib	Inlyta	AG-013736
**Tyrosine kinase inhibitor**	Nintedanib	Vargatef/Ofev	BIBF 1120
**(TKI)**	Lenvatinib	Lenvima/Lenvanix	E7080
	Cediranib	Recentin	AZD2171; NSC-732208
	Tivozanib	Fotivda	AV-951; KRN-951
	Erdafitinib	Balversa	JNJ-42756493
	Vatalanib		PTK787; ZK-222584; CGP-79787
	Apatinib (China)		YN968D1
	Anlotinib (China)		AL3818
	Fruquintinib (China)		HMPL-013
