# Supplementary material for: Cardiovascular toxicity profiles of immune checkpoint inhibitors with or without angiogenesis inhibitors: a real-world pharmacovigilance analysis based on the FAERS database from 2014 to 2022
Source: Front Immunol. 2023 May 24;14:1127128. doi: 10.3389/fimmu.2023.1127128 (PMC10244526; doi:10.3389/fimmu.2023.1127128)
Supplement: Supplementary file 3 [file DataSheet_3.pdf]

Table S11 Full list of reported cardiovascular AEs based on PTs with relevant IC025 or ROR025 for angiogenesis inhibitos alone, immune checkpoint inhibitor alone and combination therapy

**AGI alone without ICI**		**ICI alone without AGI**		**AGI combined with ICI**	
**PT**	**IC025**	**PT**	**IC025**	**PT**	**IC025**
Tumour thrombosis	3.129	Immune-mediated myocarditis	6.103	Immune-mediated myocarditis	4.107
Tumour embolism	3.083	Autoimmune myocarditis	5.429	Myocarditis	3.955
Ascites	2.502	Myocarditis	4.387	Embolism	3.120
Superior vena cava syndrome	2.236	Thrombophlebitis migrans	4.059	Autoimmune myocarditis	2.945
Thrombophlebitis migrans	2.209	Superior vena cava syndrome	3.507	Portal vein thrombosis	2.907
Portal vein thrombosis	2.136	Tumour embolism	2.978	Thrombophlebitis migrans	2.834
Hypertensive encephalopathy	2.098	Autoimmune pericarditis	2.846	Ascites	2.741
Secondary hypertension	2.074	Tumour thrombosis	2.845	Disseminated intravascular coagulation	2.534
Hypertension	2.071	Pericardial drainage	2.783	Tumour thrombosis	2.326
Embolism	2.061	Cardiac tamponade	2.677	Hypertension	2.243
Blood pressure increased	1.803	Troponin T increased	2.500	Hypertensive urgency	1.997
Vena cava thrombosis	1.733	Embolism	2.389	Troponin increased	1.867
Disseminated intravascular coagulation	1.687	Pericardial effusion	2.333	Blood pressure increased	1.857
Embolism venous	1.557	Disseminated intravascular coagulation	2.333	Blood pressure abnormal	1.654
Pulmonary artery thrombosis	1.516	Troponin increased	2.271	Cardiac dysfunction	1.471
Venous thrombosis	1.515	Troponin I increased	2.191	Splenic vein thrombosis	1.456
Venous thrombosis limb	1.495	Pulmonary artery thrombosis	1.987	Thrombotic microangiopathy	1.424
Hypertensive crisis	1.432	Lung opacity	1.976	Acute coronary syndrome	1.420
Embolism arterial	1.424	Sudden death	1.922	Pulmonary embolism	1.361
Brachiocephalic vein thrombosis	1.349	Pericardial excision	1.903	Embolism arterial	1.341
Malignant hypertension	1.306	Ascites	1.856	Venous thrombosis	1.240
Cardiac ventricular thrombosis	1.280	Atrioventricular block complete	1.842	Blood pressure fluctuation	1.188
Pulmonary tumour thrombotic microangiopathy	1.272	Blood creatine phosphokinase MB increased	1.729	Sudden death	1.170
Mesenteric vein thrombosis	1.271	Pericarditis	1.723	Blood pressure diastolic increased	1.134
Blood pressure diastolic increased	1.268	Blood creatine phosphokinase increased	1.709	Cardiac failure acute	1.117
Arrhythmia supraventricular	1.215	Superior vena cava occlusion	1.685	Embolism venous	1.004
Vena cava embolism	1.199	Myocardial necrosis marker increased	1.605	Hypertensive crisis	0.973
Ejection fraction decreased	1.187	Computerised tomogram thorax abnormal	1.575	Cardiac failure	0.944
Cardiopulmonary failure	1.184	Hypoxia	1.495	Pulmonary artery thrombosis	0.876
Splenic artery thrombosis	1.175	Cerebral infarction	1.397	Cerebral infarction	0.767
Cerebral ischaemia	1.144	Pleuropericarditis	1.302	Transient ischaemic attack	0.706
Thrombotic microangiopathy	1.121	Stress cardiomyopathy	1.298	Pericardial effusion	0.664
Jugular vein thrombosis	1.076	Cardiomyopathy	1.253	Cerebral ischaemia	0.660
Portal vein occlusion	1.049	Subclavian artery occlusion	1.108	Hypertensive encephalopathy	0.637
Myocardial injury	1.005	Vena cava thrombosis	1.071	Paraparesis	0.635
Left ventricular dysfunction	0.980	Pulmonary embolism	0.968	Mesenteric vein thrombosis	0.616
Arterial thrombosis	0.968	Supraventricular tachycardia	0.953	N-terminal prohormone brain natriuretic peptide increased	0.606
Systolic hypertension	0.966	Atrioventricular block	0.939	Hypoxia	0.582
Dyspnoea exertional	0.955	Cardiac failure acute	0.916	Cardiomyopathy	0.515
Pulmonary embolism	0.911	Paraplegia	0.890	Myocardial necrosis marker increased	0.443
Mesenteric artery thrombosis	0.895	Pulmonary venous thrombosis	0.873	Acute myocardial infarction	0.442
Retinal vein occlusion	0.889	Atrial flutter	0.855	Left ventricular dysfunction	0.439
Acute coronary syndrome	0.848	Pulmonary artery occlusion	0.854	Mental status changes	0.413
Cardiac ventricular scarring	0.842	Subendocardial ischaemia	0.840	Blood creatine phosphokinase increased	0.366
Cardiotoxicity	0.806	Cerebral ischaemia	0.834	Ischaemic stroke	0.351
Visual acuity reduced transiently	0.795	Portal vein thrombosis	0.830	Retinal vein occlusion	0.348
Venoocclusive liver disease	0.749	Thrombotic thrombocytopenic purpura	0.799	Deep vein thrombosis	0.312
Sudden death	0.735	Acute coronary syndrome	0.794	Thrombotic thrombocytopenic purpura	0.254
Cardiac dysfunction	0.734	Combined pulmonary fibrosis and emphysema	0.781	Pericardial disease	0.233
ECG signs of myocardial infarction	0.720	Venous thrombosis limb	0.748	Secondary hypertension	0.227
Device related thrombosis	0.717	Monoparesis	0.748	Embolic cerebral infarction	0.220
Aortic thrombosis	0.710	Cardiac failure	0.739	Venous thrombosis limb	0.184
Hypertensive urgency	0.706	Arterial thrombosis	0.728	Atrial enlargement	0.157
Cerebral infarction	0.698	Left ventricular dysfunction	0.704	Pulmonary infarction	0.155
Computerised tomogram thorax abnormal	0.653	Pericardial disease	0.677	Coronary artery stenosis	0.121
Collateral circulation	0.646	Sinus tachycardia	0.671	Cardiac ventricular thrombosis	0.052
Pericardial effusion	0.605	Venous thrombosis	0.665	Blood creatine phosphokinase abnormal	0.040
Hemiparesis	0.561	Atrial fibrillation	0.607	Atrial fibrillation	0.025
Axillary vein thrombosis	0.554	Cardiomyopathy acute	0.588	Troponin T increased	0.001
Blood pressure fluctuation	0.544	Embolic cerebral infarction	0.576		
Blood pressure abnormal	0.538	Hemiparesis	0.575		
Thrombotic thrombocytopenic purpura	0.535	Mesenteric vein thrombosis	0.566		
Renal artery occlusion	0.526	Carditis	0.526		
Oedema peripheral	0.526	Jugular vein thrombosis	0.523		
Congestive cardiomyopathy	0.512	Blood creatine phosphokinase abnormal	0.511		
Arteriospasm coronary	0.494	Device related thrombosis	0.511		
Renal vein thrombosis	0.490	Sinus node dysfunction	0.487		
Cardiomyopathy	0.483	Cardiopulmonary failure	0.461		
Blood pressure systolic increased	0.468	Splenic infarction	0.431		
Subclavian vein thrombosis	0.467	Autoimmune heparin-induced thrombocytopenia	0.427		
Splenic vein thrombosis	0.459	Chest X-ray abnormal	0.415		
Electrocardiogram change	0.445	Pericarditis constrictive	0.414		
Retinal artery occlusion	0.437	Acute pulmonary oedema	0.407		
Cardiac failure acute	0.412	Amaurosis	0.380		
Central venous pressure increased	0.400	Mental status changes	0.376		
Deep vein thrombosis	0.382	Oedema peripheral	0.364		
Retinal vein thrombosis	0.371	Pulmonary oedema	0.318		
Rhythm idioventricular	0.365	Acute myocardial infarction	0.312		
Oedema	0.351	Ischaemic stroke	0.302		
Ventricular hypokinesia	0.332	Cardiogenic shock	0.302		
Transient ischaemic attack	0.306	Conduction disorder	0.284		
Cardiac failure	0.302	Ventricular tachycardia	0.284		
Hemiplegia	0.301	Ejection fraction decreased	0.278		
Diastolic hypertension	0.287	Bundle branch block	0.271		
Retinopathy hypertensive	0.286	Orthostatic hypertension	0.267		
Thrombosis	0.234	Dyspnoea	0.255		
Pelvic venous thrombosis	0.233	Oedema	0.214		
Blood creatine phosphokinase MB increased	0.227	Arrhythmia supraventricular	0.190		
Troponin T increased	0.212	Paraparesis	0.179		
Ventricular dysfunction	0.206	Thrombophlebitis	0.177		
Catheter site thrombosis	0.201	Renal infarct	0.151		
Paget-Schroetter syndrome	0.175	Central venous catheterisation	0.148		
Hypertensive emergency	0.173	Ventricular arrhythmia	0.130		
Hypoxia	0.172	Subclavian vein thrombosis	0.112		
Coronary artery stenosis	0.148	Lacunar infarction	0.095		
Lung opacity	0.143	Dyspnoea exertional	0.052		
Choroidal infarction	0.138	Cerebellar infarction	0.045		
Mental status changes	0.128	Jugular vein occlusion	0.041		
Hepatic infarction	0.120	Embolism venous	0.034		
Dyspnoea	0.111	Monoplegia	0.028		
Pulmonary thrombosis	0.100	Thrombotic stroke	0.020		
Thrombophlebitis	0.061	Congestive hepatopathy	0.003		
Left atrial dilatation	0.058				
Pulmonary oedema	0.043				
Troponin increased	0.027				
Hepatic artery embolism	0.015				
Labile blood pressure	0.009				
Chest pain	0.008				
Renal vascular thrombosis	0.001				

**AGI alone without ICI**		**ICI alone without AGI**		**AGI combined with ICI**	
PT	**ROR025**	**PT**	**ROR025**	**PT**	**ROR025**
Tumour thrombosis	8.573	Autoimmune myocarditis	36.160	Myocarditis	15.920
Tumour embolism	8.313	Immune-mediated myocarditis	35.596	Immune-mediated myocarditis	12.092
Ascites	5.687	Myocarditis	20.961	Embolism	9.028
Superior vena cava syndrome	4.830	Thrombophlebitis migrans	16.212	Autoimmune myocarditis	8.052
Thrombophlebitis migrans	4.747	Superior vena cava syndrome	11.580	Portal vein thrombosis	7.958
Portal vein thrombosis	4.463	Tumour embolism	8.090	Thrombophlebitis migrans	7.789
Hypertensive encephalopathy	4.413	Tumour thrombosis	7.433	Ascites	6.852
Secondary hypertension	4.361	Pericardial drainage	7.182	Disseminated intravascular coagulation	6.023
Embolism	4.217	Cardiac tamponade	6.514	Tumour thrombosis	5.661
Hypertension	4.211	Troponin T increased	5.865	Hypertension	4.785
Blood pressure increased	3.500	Embolism	5.330	Hypertensive urgency	4.619
Vena cava thrombosis	3.414	Disseminated intravascular coagulation	5.113	Troponin increased	3.888
Disseminated intravascular coagulation	3.256	Pericardial effusion	5.094	Blood pressure increased	3.675
Embolism venous	3.004	Troponin increased	4.923	Blood pressure abnormal	3.273
Pulmonary artery thrombosis	2.983	Troponin I increased	4.744	Splenic vein thrombosis	3.255
Venous thrombosis	2.917	Pulmonary artery thrombosis	4.198	Cardiac dysfunction	3.048
Venous thrombosis limb	2.894	Lung opacity	4.094	Embolism arterial	2.923
Embolism arterial	2.779	Pericardial excision	4.057	Thrombotic microangiopathy	2.855
Brachiocephalic vein thrombosis	2.744	Sudden death	3.864	Acute coronary syndrome	2.854
Hypertensive crisis	2.733	Atrioventricular block complete	3.673	Pulmonary embolism	2.625
Pulmonary tumour thrombotic microangiopathy	2.601	Ascites	3.664	Venous thrombosis	2.599
Malignant hypertension	2.583	Superior vena cava occlusion	3.548	Blood pressure diastolic increased	2.416
Cardiac ventricular thrombosis	2.530	Blood creatine phosphokinase MB increased	3.521	Sudden death	2.411
Mesenteric vein thrombosis	2.519	Pericarditis	3.362	Blood pressure fluctuation	2.371
Vena cava embolism	2.493	Blood creatine phosphokinase increased	3.314	Cardiac failure acute	2.351
Blood pressure diastolic increased	2.462	Myocardial necrosis marker increased	3.190	Pulmonary artery thrombosis	2.241
Splenic artery thrombosis	2.459	Computerised tomogram thorax abnormal	3.163	Embolism venous	2.240
Arrhythmia supraventricular	2.428	Hypoxia	2.855	Hypertensive crisis	2.094
Cardiopulmonary failure	2.328	Pleuropericarditis	2.725	Cardiac failure	1.976
Ejection fraction decreased	2.306	Cerebral infarction	2.674	Hypertensive encephalopathy	1.966
Portal vein occlusion	2.269	Stress cardiomyopathy	2.538	Mesenteric vein thrombosis	1.875
Cerebral ischaemia	2.262	Subclavian artery occlusion	2.478	Paraparesis	1.821
Thrombotic microangiopathy	2.212	Cardiomyopathy	2.434	N-terminal prohormone brain natriuretic peptide increased	1.817
Myocardial injury	2.204	Vena cava thrombosis	2.235	Cerebral infarction	1.788
Jugular vein thrombosis	2.187	Pulmonary venous thrombosis	2.127	Cerebral ischaemia	1.766
Systolic hypertension	2.090	Pulmonary artery occlusion	2.124	Transient ischaemic attack	1.707
Arterial thrombosis	2.029	Subendocardial ischaemia	2.106	Pericardial effusion	1.674
Left ventricular dysfunction	2.013	Combined pulmonary fibrosis and emphysema	2.020	Myocardial necrosis marker increased	1.624
Mesenteric artery thrombosis	2.002	Autoimmune pericarditis	2.005	Pericardial disease	1.571
Dyspnoea exertional	1.957	Supraventricular tachycardia	1.992	Hypoxia	1.567
Retinal vein occlusion	1.917	Atrioventricular block	1.979	Secondary hypertension	1.564
Pulmonary embolism	1.891	Pulmonary embolism	1.975	Cardiomyopathy	1.534
Visual acuity reduced transiently	1.876	Cardiac failure acute	1.953	Left ventricular dysfunction	1.500
Acute coronary syndrome	1.836	Paraplegia	1.947	Retinal vein occlusion	1.493
Cardiotoxicity	1.784	Portal vein thrombosis	1.872	Atrial enlargement	1.493
Cardiac ventricular scarring	1.783	Atrial flutter	1.866	Embolic cerebral infarction	1.479
Hypertensive urgency	1.777	Cerebral ischaemia	1.859	Acute myocardial infarction	1.429
ECG signs of myocardial infarction	1.756	Pericardial disease	1.844	Mental status changes	1.407
Collateral circulation	1.750	Thrombotic thrombocytopenic purpura	1.827	Thrombotic thrombocytopenic purpura	1.380
Device related thrombosis	1.733	Monoparesis	1.792	Blood creatine phosphokinase abnormal	1.380
Aortic thrombosis	1.727	Acute coronary syndrome	1.790	Blood creatine phosphokinase increased	1.366
Venoocclusive liver disease	1.727	Venous thrombosis limb	1.785	Venous thrombosis limb	1.358
Cardiac dysfunction	1.718	Arterial thrombosis	1.767	Ischaemic stroke	1.356
Sudden death	1.698	Cardiomyopathy acute	1.761	Pulmonary infarction	1.331
Computerised tomogram thorax abnormal	1.665	Left ventricular dysfunction	1.690	Cardiac ventricular thrombosis	1.318
Cerebral infarction	1.643	Cardiac failure	1.687	Deep vein thrombosis	1.287
Renal artery occlusion	1.606	Carditis	1.665	Troponin T increased	1.272
Axillary vein thrombosis	1.604	Venous thrombosis	1.663	Coronary artery stenosis	1.258
Pericardial effusion	1.543	Embolic cerebral infarction	1.644	Hypertensive emergency	1.249
Central venous pressure increased	1.531	Sinus tachycardia	1.637	Splenic infarction	1.217
Renal vein thrombosis	1.516	Blood creatine phosphokinase abnormal	1.629	Intracardiac thrombus	1.144
Thrombotic thrombocytopenic purpura	1.501	Mesenteric vein thrombosis	1.627	Electrocardiogram T wave abnormal	1.134
Hemiparesis	1.499	Device related thrombosis	1.560	Cardiotoxicity	1.092
Splenic vein thrombosis	1.493	Jugular vein thrombosis	1.547	Myocardial ischaemia	1.073
Blood pressure fluctuation	1.477	Pericarditis constrictive	1.543	ECG signs of myocardial infarction	1.056
Blood pressure abnormal	1.474	Atrial fibrillation	1.539	Haemorrhagic stroke	1.056
Congestive cardiomyopathy	1.470	Autoimmune heparin-induced thrombocytopenia	1.528	Atrial fibrillation	1.052
Subclavian vein thrombosis	1.463	Hemiparesis	1.526	Sinus tachycardia	1.038
Electrocardiogram change	1.463	Sinus node dysfunction	1.494	Oedema peripheral	1.029
Arteriospasm coronary	1.460	Amaurosis	1.453	Vena cava thrombosis	1.004
Oedema peripheral	1.450	Splenic infarction	1.452		
Rhythm idioventricular	1.432	Cardiopulmonary failure	1.451		
Cardiomyopathy	1.424	Chest X-ray abnormal	1.420		
Retinal artery occlusion	1.413	Acute pulmonary oedema	1.388		
Diastolic hypertension	1.409	Orthostatic hypertension	1.366		
Retinal vein thrombosis	1.406	Bundle branch block	1.348		
Blood pressure systolic increased	1.406	Mental status changes	1.327		
Cardiac failure acute	1.367	Conduction disorder	1.322		
Retinopathy hypertensive	1.367	Jugular vein occlusion	1.313		
Deep vein thrombosis	1.315	Oedema peripheral	1.302		
Ventricular hypokinesia	1.311	Arrhythmia supraventricular	1.275		
Catheter site thrombosis	1.301	Cardiogenic shock	1.272		
Oedema	1.289	Pulmonary oedema	1.268		
Choroidal infarction	1.277	Acute myocardial infarction	1.268		
Hemiplegia	1.263	Ischaemic stroke	1.264		
Blood creatine phosphokinase MB increased	1.260	Ventricular tachycardia	1.255		
Paget-Schroetter syndrome	1.257	Ejection fraction decreased	1.249		
Transient ischaemic attack	1.253	Paraparesis	1.227		
Cardiac failure	1.244	Renal infarct	1.213		
Pelvic venous thrombosis	1.242	Thrombophlebitis	1.200		
Troponin T increased	1.238	Dyspnoea	1.200		
Ventricular dysfunction	1.224	Subclavian vein thrombosis	1.195		
Hepatic infarction	1.213	Congestive hepatopathy	1.185		
Hypertensive emergency	1.205	Oedema	1.179		
Hepatic artery embolism	1.196	Central venous catheterisation	1.176		
Thrombosis	1.186	Mesenteric artery thrombosis	1.176		
Lung opacity	1.168	Ventricular arrhythmia	1.160		
Coronary artery stenosis	1.152	Lacunar infarction	1.157		
Renal vascular thrombosis	1.150	Thrombotic stroke	1.152		
Hypoxia	1.143	Inferior vena caval occlusion	1.141		
Ventricular dyskinesia	1.129	Cerebellar infarction	1.128		
Left atrial dilatation	1.114	Coronary angioplasty	1.113		
Hepatic vein thrombosis	1.114	Embolism venous	1.092		
Mental status changes	1.110	Monoplegia	1.080		
Pulmonary thrombosis	1.097	Respiratory fatigue	1.074		
Thrombophlebitis	1.087	Dyspnoea exertional	1.058		
Dyspnoea	1.084	Prinzmetal angina	1.054		
Labile blood pressure	1.080	Cardiotoxicity	1.038		
Troponin increased	1.052	Peripheral artery thrombosis	1.006		
Pulmonary oedema	1.043				
Paroxysmal arrhythmia	1.035				
Lacunar infarction	1.033				
Accelerated hypertension	1.033				
Blood pressure inadequately controlled	1.027				
Myocardial ischaemia	1.024				
Orthopnoea	1.015				
Left ventricular failure	1.015				
Chest pain	1.012				
Ventricular hypertrophy	1.003				
Monoplegia	1.001				
